# Switch Strategy from Direct Aspiration First Pass Technique to Solumbra Improves Technical Outcome in Endovascularly Treated Stroke

**DOI:** 10.3390/ijerph18052670

**Published:** 2021-03-06

**Authors:** Enrico Pampana, Sebastiano Fabiano, Gianluca De Rubeis, Luca Bertaccini, Alessandro Stasolla, Alberto Pingi, Valeria Cozzolino, Marilena Mangiardi, Sabrina Anticoli, Claudio Gasperini, Enrico Cotroneo

**Affiliations:** 1 Department of Diagnostic, UOC of Neuroradiology and Interventional Neuroradiology, San Camillo-Forlanini Hospital, 00152 Rome, Italy; sebastianofabiano@gmail.com (S.F.); lucabertaccini84@gmail.com (L.B.); alestaso@tiscali.it (A.S.); albepingi@yahoo.it (A.P.); valeria_cozzolino@yahoo.it (V.C.); cotro52@libero.it (E.C.); 2 Emergency Department, UOSD, Stroke Unit, San Camillo-Forlanini Hospital, 00152 Rome, Italy; marilenamangiardi@gmail.com (M.M.); sabrina.anticoli@gmail.com (S.A.); 3 Department of Neuroscience, UOC of Neurology, San Camillo-Forlanini Hospital, 00152 Rome, Italy; c.gasperini@libero.it

**Keywords:** stroke, cerebrovascular, thrombolysis, mechanical, thrombectomy, aspiration, outcome, treatment

## Abstract

Background: The major endovascular mechanic thrombectomy (MT) techniques are: Stent-Retriever (SR), aspiration first pass technique (ADAPT) and Solumbra (Aspiration + SR), which are interchangeable (defined as switching strategy (SS)). The purpose of this study is to report the added value of switching from ADAPT to Solumbra in unsuccessful revascularization stroke patients. Methods: This is a retrospective, single center, pragmatic, cohort study. From December 2017 to November 2019, 935 consecutive patients were admitted to the Stroke Unit and 176/935 (18.8%) were eligible for MT. In 135/176 (76.7%) patients, ADAPT was used as the first-line strategy. SS was defined as the difference between first technique adopted and the final technique. Revascularization was evaluated with modified Thrombolysis In Cerebral Infarction (TICI) with success defined as mTICI ≥ 2b. Procedural time (PT) and time to reperfusion (TTR) were recorded. Results: Stroke involved: Anterior circulation in 121/135 (89.6%) patients and posterior circulation in 14/135 (10.4%) patients. ADAPT was the most common first-line technique vs. both SR and Solumbra (135/176 (76.7%) vs. 10/176 (5.7%) vs. 31/176 (17.6%), respectively). In 28/135 (20.7%) patients, the mTICI was ≤ 2a requiring switch to Solumbra. The vessel’s diameter positively predicted SS result (odd ratio (OR) 1.12, confidence of interval (CI) 95% 1.03–1.22; *p* = 0.006). The mean number of passes before SS was 2.0 ± 1.2. ADAPT to Solumbra improved successful revascularization by 13.3% (107/135 (79.3%) vs. 125/135 (92.6%)). PT was superior for SS comparing with ADAPT (71.1 min (CI 95% 53.2–109.0) vs. 40.0 min (CI 95% 35.0–45.2); *p* = 0.0004), although, TTR was similar (324.1 min (CI 95% 311.4–387.0) vs. 311.4 min (CI 95% 285.5–338.7); *p* = 0.23). Conclusion: Successful revascularization was improved by 13.3% after switching form ADAPT to Solumbra (final mTICI ≥ 2b was 92.6%). Vessel’s diameter positively predicted recourse to SS.

## 1. Introduction

Between 2015 and 2016, six randomized control trials (MR CLEAN, ESCAPE, EXTEND-IA, SWIFT-PRIME, REVASCAT and THRACE trials) showed positive results in favor of mechanical thrombectomy (MT) for acute stroke management [1], confirmed by the meta-analysis of the HERMES collaboration (odd ratio (OR) 2.49 (confidence of interval (CI) 95% 1.76–3.53) for reducing disability at 90 days) [2]. In 2019, the COMPASS trial [3] (non-inferiority design) showed equivalence outcome between Stent-Retriever (SR) and aspiration first pass technique (ADAPT) for mRS = 0–2 (52% (CI 95% 43.8–60.3) vs. 50% (CI 95% 41.6–57.4); respectively, *p* = 0.0014). Considering this new evidence, the 2019 AHA/ASA update guideline for stroke management assigned to ADAPT a level of I B-R (moderate quality of evidence) [4]. In addition, SNIS Standards and Guidelines Committee [5] confirmed MT guidelines for posterior circulation stroke. 

In 2013, Kang et al. [6] codified the concept of a switching strategy to maximize the technical outcome, defined as the change from one EVT technique to another after angiographic recanalization failure acording to guidelines [4,7], the technical goal for thrombectomy was modified Thrombolysis in Cerebral Infarction (mTICI) of grade 2b/3 to increase the probability of a good functional clinical outcome [8]. According to a survey in 2016, ADAPT was the most used technique in the US [9], but it was associated with a higher rescue technique requirement (OR 1.367 (CI 95% 1.019–1.834)) [10]. Stapleton et al. [11] demonstrated that there is no difference in times between ADAPT and ADAPT + stent retriever for rescue, although, ADAPT had a faster procedural time comparing with SR [11]. In addition, the sequential endovascular thrombectomy approach (ADAPT followed by SR) led to an overall cost-saving of €2747.28 (approximately $3235.66) [12]. 

The aim of our study was to evaluate the impact of switching from ADAPT to Solumbra on the successful revascularization in a hub center for cerebrovascular disease treatment. 

## 2. Materials and Methods

This study was approved by the institutional review board on 21 April 2020 (Committee: “Lazio 1”, identification code: OssR186, protocol number: 458). Informed consent was waived due to the retrospective nature of this study. The manuscript was drafted according to STROBE guidelines [13].

**Figure 1:**
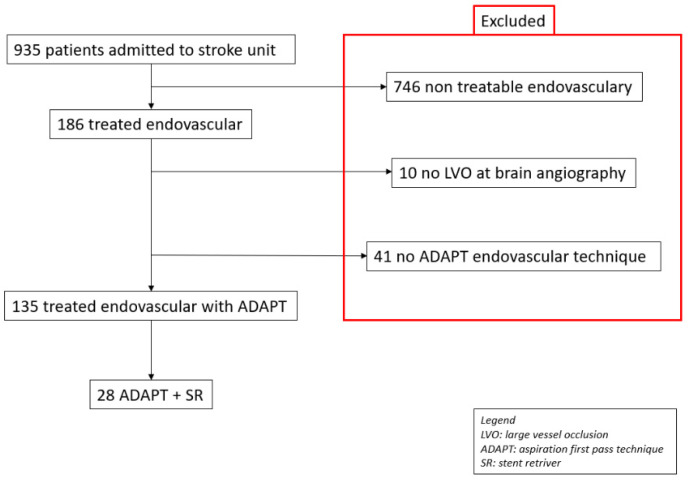
Flowchart of the study.

This is a retrospective, single center, cohort study. From December 2017 (starting 24 h service) to November 2019, 935 consecutive patients were admitted to the Stroke Unit of a tertiary/hub center. Among these, 186/935 (19.9%) were eligible for endovascular treatment according to a multidisciplinary meeting (strokologist and interventional neuroradiologist), which evaluated each clinical case based on current guidelines [4,7]. The reasons for ineligibility for MT were: Hemorrhagic stroke, clinical onset > 6 h or no imaging mismatch, Alberta Stroke Program Early Computed Tomography Score of <6, and non-large vessel occlusion (LVO) [4]. The clinical details of the cohort are displayed in Table 1. The exclusion criterion for this study was the absence of intra-cranial vascular occlusion at brain angiography (10/186 [5.4%]) and the use of MT techniques other than an ADAPT (41/176 (23.3%), including 10/41 (24.4%) stent retriever and 31/41 (75.6%) Solumbra) [14,15,16]. The final population was composed by 135/935 (14.4%). In particular, the study was focused on the subgroup of the switching strategy (SS) from ADAPT to Solumbra (28/135 (20.7%)) (Figure 1). Since there is no definitive indication regarding which endovascular technique should be used for thrombectomy [4], the choice was deemed to the interventional neuroradiologist at the time of the procedure. The switching strategy was defined as the difference between the initial and final technique [6]. As there is no indication of the number of attempts before changing the initial technique, the decision for SS was leaved to the interventional neuroradiologist. The revascularization was evaluated according to the guideline with mTICI, and mTICI = 2b or 3 was defined as successful [4,7]. In addition, procedural time and time to reperfusion was recorded for the entire cohort and for the two subgroups (ADAPT and Solumbra).

### 2.1. Endovascular Techniques 

The procedures were performed by five experienced (all at least >7 years) neuroradiologists in a dedicated angio-suite (biplane Artis Zee, Siemens, Erlangen, Germany). 

All procedures were performed using arterial access under sedation or general anesthesia according to present clinical status and stroke severity. In 130/135 (96.3%) femoral access was obtained with a 6F long vascular sheath (Neuro max 088; Penumbra Inc., Alameda, CA, USA) or AXS Infinity LS (Stryker Neurovascular, Fremont, CA, USA) advanced in the internal carotid or in the vertebral artery, according to previous imaging findings; an angiogram of the culprit vessel was also performed. In the remaining 5/135 (3.7%) patients, the femoral access was not achievable; therefore, in 3/5 (60%) a trans-brachial approach was performed and in 2/5 (40%) cases a direct common carotid puncture was needed because of anatomical issues: In these cases the access to cerebral circulation was gained directly by means of 6 Fr intermediate catheter.

The ADAPT [15,17,18] was performed bi-axial or tri-axial, depending on the size of the occluded vessel, both with Penumbra (ACE68 and 3MAX; Penumbra Inc., Alameda, CA, USA) or Stryker Neurovascular (AXS Catalyst 6 and AXS Offset; Stryker Neurovascular, Fremont, CA, USA) systems. 

The Solumbra technique as rescue switching strategy [16] was performed advancing a stent-retriever (Trevo Retriever; Stryker Neurovascular, Fremont, CA, USA) or Solitare (Covidien, Mansfield, MA, USA) through the intermediate aspiration catheter used for ADAPT. 

### 2.2. Study Outcomes

The primary outcome was the improvement of revascularization as measured by mTICI in the subgroup of switching strategy. The mTICI was treated as binary outcome: unsuccessful (mTICI ≤ 2a) and successful (mTICI ≥ 2b) revascularization.

The secondary outcome was to evaluate the prognostic factors associated with the switching strategy.

The tertiary outcome was the procedural time and the time to reperfusion in the two subgroups (ADAPT/Solumbra).

### 2.3. Database Preparation 

Clinical data were retrospectively derived from a prospective database drafted by the Stroke Unit. The radiological parameters were evaluated through RIS/PACS systems by two neuroradiologists (>5 years of experience), not involved in the procedure, in consensus. 

### 2.4. Statistical Analysis

The Kolmogorov-Smirnov Z test was performed to assess the normality distribution for all variables tested. Continuous normal variables were expressed as mean ± standard deviation. Continuous non-normal variables were expressed as median and confidential interval (CI) 95%. The chi-square test was used for comparing the usage rate of the techniques at the two time points (first-line vs. final technique). The mTICI score was classified in a binary outcome: Unsuccessful (mTICI = 1 or 2a) or successful (mTICI = 2b or 3) revascularization. Since mTICI was categorized as binary, logistic regression (univariate and multivariate) was performed to predict the switch strategy and successful revascularization. In particular, if a parameter was statistically significant at univariate logistic regression, it was included in the multivariate logistic regression. Mann-Whitney test was used for comparing procedural time and time to reperfusion between the ADAPT and the SS cohorts. Statistical analysis was performed, and the graph was plotted using MedCalc 18.2.1 software (MedCalc, Mariakerke, Belgium). *p* values < 0.05 were considered statistically significant, and all *p* values were calculated using a two-tailed significance level.

## 3. Results

One-hundred-eighty-six cerebral angiographies were performed. In 10/186 (5.4%) patients, intracranial LVO was no longer observed at the pre-procedural digital subtracted angiography and, therefore, they were excluded from the analysis. In 10/176 (5.7%) and 31/176 (17.6%) patients, a stent retriever and Solumbra were used respectively as first-line techniques and excluded from further analysis. The final population of ADAPT as initial technique encompassed 135/186 (72.6%) patients. 

The anterior circulation was involved in 121/135 (89.6%), in particular, M1 segment was involved in 102/135 (75.6%), M2 in 18/135 (13.3%) and A1 in 1/135 (0.7%). The posterior one in 14/135 (10.4%). Among the subgroup of SS, 22/28 (78.6%) was M1 segment, 2/28 (7.1%) was M2 segment and 4/28 (14.3%) was posterior circulation. The wake-up stroke rate was 20/135 (14.8%). The median time of symptoms-to-groin puncture was 4.45 h (CI 95% 4.19–5.00).

In 28/135 (20.7%), the first-line ADAPT presented and unsuccessful recanalization (mTICI ≤ 2a) requiring to switch to the Solumbra technique by employing a stent retriever. The only factor that positively predicted a switching strategy was the vessel’s diameter (odd ratio (OR) 1.12, CI 95% 1.03–1.22; *p* = 0.006) (the greater the diameter, the higher the chance to resort to SS) (Table 2.). Switch strategy from ADAPT to Solumbra improved the mTICI ≥ 2b in 18/28 (64.3%). The median number of passes was 2.0 (CI 95% 1.0–2.0, mean 2.0 ± 1.2) for the complete series and 3.0 (CI 95% 2.0–3.0, mean 2.9 ± 1.2) for the SS subgroup. 

**Figure 2:**
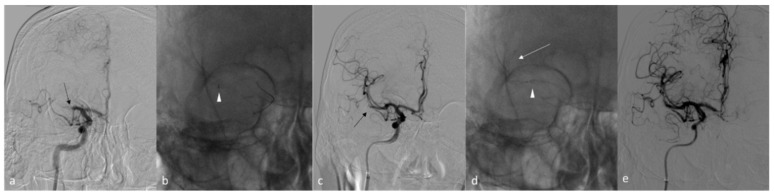
Clinical Case. Right M1/M2 ischemic stroke with a time to groin puncture of 135 min. (**a**) showed occlusion of right M1 tract of middle cerebral artery (black arrow), treated by mechanical thrombectomy (**b**) using aspiration first pass technique (arrowhead); since revascularization was considered unsuccessful after 2 passes (mTICI = 2a) for the persistence occlusion of M1/M2 (black arrow) (**c**), a switch to Solumbra technique was performed with aspiration catheter (arrowhead) at M1 tract and stent-retriever at M1/M2 (arrow) (**d**). The digital subtracted angiography at the end of the procedure (**e**) showed successful revascularization (mTICI = 3).

ADAPT to Solumbra improved mTICI ≥ 2b rate by 13.3% (107/135 (79.3%) vs. 125/135 (92.6%). In particular, final mTICI scores were: 1 in 3/135 (2.2%), 2a in 7/135 (5.2%), 2b in 42/135 (31.1%) and 83/135 (61.5%) (Figure 2.). 

The median procedural time was 43.1 min (CI 95% 36.4–48.5, average 58.1 ± 46.8), the ADAPT subgroup was significantly faster comparing with the SS one (40.0 min (CI 95% 35.0–45.2) vs. 71.1 min (CI 95% 53.2–109.0); *p* = 0.0004). However, no differences were found in time to reperfusion between the two subgroups (ADAPT vs. SS; 311.4 min (CI 95% 285.5–338.7) vs. 324.1 min (CI 95% 311.4–387.0); *p* = 0.23).

## 4. Discussion

Switching from ADAPT to Solumbra, after two attempts, led to a successful revascularization improvement of 13.3%, vessel’s diameter positively predicted the switching strategy (OR 1.12, CI 95% 1.03–1.22). 

ADAPT technique has experienced a growing usage rate in most stroke centers due to its simplicity and inexpensiveness as confirmed by a US survey (ADAPT vs. SR vs. Solumbra 39.7% vs. 28.2% vs. 28.2%, respectively) [9]. The switching strategy was first formalized by Kang et al. [6] for anterior circulation leading to not significantly better angiographic outcome (only ADAPT vs. ADAPT to SR: 73.8% vs. 85.1%; *p* = 0.10). Due to increased recourse to ADAPT and its high adjunctive device rate needed comparing with SR (45.2% vs. 13.5%) [17], a focus on this subset of patients is needed. In fact, in a recent metanalysis by Zhang et al. [18] demonstrated that the OR of ADAPT for adjunctive therapy vs. stent retriever was (OR 2.24, CI 95% 1.41–3.57, *p* = 0.0007, I^2^ = 83%) However, the 45.2% of adjunctive device employed rate of Lapergue et al. [17] was higher compared with the present series in which the switching rate from ADAPT to Solumbra was 20.7%. In addition, switch from ADAPT to Solumbra is the easiest and fastest rescue strategy [11], allowing a quicker next revascularization attempt. In fact, in the paradigm of “time is brain”, speed is fundamental for increasing chance for good clinical outcome. This concept is sustained by the similar time to reperfusion between ADAPT and Solumbra rescue (311.4 min (CI 95% 285.5–338.7) vs. 324.1 min (CI 95% 311.4–387.0); *p* = 0.23). Moreover, Stapleton et al. [11] demonstrated that ADAPT was faster than Stent Retriever in achieving good recanalization. 

The median number of passes of the present series was 2.0 ± 1.2 which was in line with existing literature (range 1.3–1.9) [17]. However, the average number of passes increased in the switching strategy subgroup to 2.9 ± 1.2, this evidence was consistent with ASTER trial protocol in which the minimum attempts before switch to another MT technique was three [19,20]. No single clinical parameter was able to predict the switching strategy as shown also by another study [17]; on the contrary, the diameter of vessels significantly predicted the switching strategy (OR 1.12, CI 95% 1.03–1.22, *p* = 0.006) and, therefore, a failure of the first-line technique (Table 2.). This finding was is contrast with Shaarada et al. [21] who demonstrated, in a M1-stroke cohort, that large vessel diameter (*p* = 0.001) positive predicted first pass effect. This discrepancy could be explained by the difference in the outcome between the studies (switching strategy and first pass effect) and by a different distribution of occlusion sites. In fact, Blanc et al. [22] demonstrated that ADAPT was efficient in Middle Cerebral Artery circulation for an optimal vessel size and aspiration-catheter-diameter-ratio, while Mönch et al. [23] demonstrated that vessel diameter did not predict a good functional outcome (OR 1.2, CI 95% 0.28–5.26, *p* = 0.659). Anyhow, the present series was mostly on M1 tract of MCA (literature reported average diameter 2.3 mm) [24] and the aspiration catheter used were ACE68 (inner diameter 1.73 mm) and Catalyst 6 (inner diameter 1.52 mm), the vessel/catheter diameter ratio of ≤1.51 (best probability of achieving mTICI ≤ 2b) was respected [25,26].

The recourse to SS improving technical success rate of 13.3% leading to mTICI ≥ 2b of 92.6% (125/135) (before SS, 79.3% (107/135)). This data was higher compared with those of HERMES collaboration meta-analysis (71%) (stent retriever trials only) [2], and slightly superior to COMPASS trial (81%) [3] and ASTER trial (85.4%) [aspiration-technique trials] [19]. Although, the nature of the studies was different (randomized controlled trial (RCT) vs. observational study), the greater similarity with the two aspiration-technique trials comparing with HERMES collaboration reflected the consistency of our data while in a real-world setting [27]. In addition, the rate of mTICI ≥ 2b is positively correlated with functional independence [28]. 

Mean procedural time of the entire cohort (ranging from 21 to 75.5 min) and of the SS subgroup (ranging from 53 to 68 min) was similar with other series reported in literature [11,29]. Moreover, although, the procedural time was significantly inferior in ADAPT comparing with the SS subgroups (40.0 min (CI 95% 35.0–45.2) vs. 71.1 min (CI 95% 53.2–109.0); *p* = 0.0004), the time to reperfusion was similar (311.4 min (CI 95% 285.5–338.7) vs. 324.1 min (CI 95% 311.4–387.0), *p* = 0.23) and in line with MR CLEAN trial (340 min (interquartile range (IQR) 274–395)) [30]. This evidence suggested that procedural time had only a limited impact on reperfusion time (symptoms to reperfusion), which is one the most important prognostic parameter in stroke therapy [30]. For this reason, much more efforts must be employed for decreasing pre-procedural time. 

Therefore, in a setting of ADAPT first-line strategy, for increasing chance to obtain a successful revascularization and keeping low the time to reperfusion, it seems to be reasonable to switch to Solumbra after failure (mTICI ≤ 2a) of maximum three attempts of MT. 

Our study presented several limitations. Firstly, the nature of the study was a retrospectively non-randomized, single-arm, single-center study. Secondly, the study population was not-homogeneous especially regarding the vessel occlusion site (e.g., anterior vs. posterior circulations). Thirdly, the first-line endovascular technique used and the decision to switch technique were not standardized and left to the surgeon at the moment of the procedure. Quarterly, 10/186 (5.4%) were excluded from the analysis due to the absence of LVO eat the pre-procedural digital subtracted angiography.

## 5. Conclusions

Switching strategy from ADAPT to Solumbra after reperfusion failure improved the rate of successful revascularization by 13.3% leading to mTICI ≥ 2b in 92.6% patients (107/135 (79.3%) vs. 125/135 (92.6%)). The procedural time was significantly higher in switching strategy subgroup comparing with ADAPT group, but the time to reperfusion was similar (ADAPT vs. SS; 311.4 min (CI 95% 285.5–338.7) vs. 324.1 min (CI 95% 311.4–387.0); *p* = 0.23). The diameter of vessel positively predicts the necessity to apply a switching strategy.

## Figures and Tables

**Table 1:** Clinical details.

	ADAPT Treated (*n* = 135)
Age (years) (median and IC 95%)	72.0 (70.0–75.0)
Male (*n* %)	68 (50.7%)
BMI (median and IC 95%)	25.1 (24.7–26.4)
Familiar History (*n* %)	24 (17.8%)
Smoking (*n* %)	32 (23.7%)
Atrial fibrillation (*n* %)	38 (28.1%)
Arterial Hypertension (*n* %)	77 (57.0%)
Obesity (*n* %)	22 (16.3%)
Prior TIA (*n* %)	2 (1.5%)
Total Cholesterol (median and IC 95%)	154.0 (147.4–166.6)
HDL (median and IC 95%)	43.5 (41.0–45.3)
LDL (median and IC 95%)	104.5 (91.7–114.1)
NIHSS at time of admission	16 (14.8–18.0)
NIHSS at time of demission	4 (2.0–7.0)
Intravenous fibrinolytic	61 (45.2%)

BMI: body mass index, TIA: transient ischemic attack, HDL: high-density lipoprotein, LDL: low-density lipoprotein, NIHSS: National Institutes of Health Stroke Scale.

**Table 2:** Logistic regression for switching strategy.

	Univariate OR (CI 95%)	*p*
Age	1.03 (0.99–1.06)	0.12
Male	0.80 (0.35–1.85)	0.22
BMI	0.99 (0.95–1.04)	0.80
Total cholesterol	0.99 (0.97–1.01)	0.17
LDL cholesterol	1.00 (1.0–1.01)	0.23
Smoking	1.39 (0.44–4.41)	0.58
Arterial hypertension	1.32 (0.43- 4.01)	0.63
NOA	1.03 (0.20–5.3)	0.97
NIHSS	1.05 (0.96–1.14)	0.28
Wake up stroke	0.95 (0.29–3.10)	0.92
Time from symptoms to groin puncture (min)	1.05 (0.90–1.23)	0.55
Posterior circulation	0.61 (0.13–2.89)	0.53
Vessel’s diameter (mm)	1.12(1.03–1.22)	0.006

OR: odd ratio; CI: confidence interval; BMI: body mass index; LDL: low-density lipoprotein; NOA: new oral anticoagulant; NIHSS: National Institute of Health Stroke Score.

## Data Availability

The data that support the findings of this study are available from the corresponding author, upon reasonable request.
